# The prognostic value of dobutamine stress echocardiography amongst British Indian Asian and Afro-Caribbean patients: a comparison with European white patients

**DOI:** 10.1186/s12947-015-0028-1

**Published:** 2015-08-06

**Authors:** Jamie M. O’Driscoll, Claire Rossato, Paula Gargallo-Fernandez, Marco Araco, Dimitrios Giannoglou, Sanjay Sharma, Rajan Sharma

**Affiliations:** Department of Cardiology, St George’s Healthcare NHS Trust, Blackshaw Road, Tooting, SW17 0QT London; St George’s, University of London, Cranmer Terrace, SW17 0RE London

**Keywords:** Dobutamine stress echocardiography, Ethnicity, Ischaemia, Transthoracic echocardiography

## Abstract

**Background:**

The incidence of cardiovascular disease is considerably disparate among different racial and ethnic populations. While dobutamine stress echocardiography (DSE) has been shown to be useful in Caucasian patients, its role among ethnic minority groups remains unclear. This study aimed to investigate the prognostic importance of DSE in three ethnic groups in the UK.

**Methods:**

DSE was performed on 6231 consecutive patients. After exclusions, 5329 patients formed the study (2676 [50.2 %] Indian Asian, 2219 [41.6 %] European white and 434 [8.1 %] Afro-Caribbean). Study outcome measures were non-fatal cardiac events (NFCE) and all-cause mortality.

**Results:**

There were 849 (15.9 %) NFCE and 1365 (25.6 %) deaths over a median follow-up period of 4.6 years. In total 1174 (22 %) patients had inducible myocardial ischaemia during DSE, 859 (16.1 %) had fixed wall motion abnormalities and 3645 (68.4 %) patients had a normal study. Ethnicity did not predict events. Among the three ethnic groups, ischaemia on DSE was associated with 2 to 2.5 times the risk of non-fatal cardiac events and 1.2 to 1.4 times the risk of all-cause mortality. Peak wall motion score index was the strongest independent predictor of non-fatal cardiac events and all-cause mortality in all groups. The C statistic for the prediction of NFCE and all-cause mortality were significantly higher when DSE parameters were added to the standard risk factors for all ethnic groups.

**Conclusions:**

DSE is a strong predictor of NFCE and all-cause mortality and provides predictive information beyond that provided by standard risk factors in three major racial and ethnic groups. No major differences among racial and ethnic groups in the predictive value of DSE was detected.

**Electronic supplementary material:**

The online version of this article (doi:10.1186/s12947-015-0028-1) contains supplementary material, which is available to authorized users.

## Background

The UK has become more ethnically diverse with an increase in both the number and proportion of Afro-Caribbean and Asian minority ethnic groups. In 2011, all Afro-Caribbean and Asian minority groups accounted for 13% of the population in England and Wales [1]. The incidence of cardiovascular disease (CVD) is considerably disparate among different racial and ethnic populations [2]. CVD mortality is 40 % higher in Indian Asians compared with European white groups [3] and is significantly greater among Afro-Caribbeans compared with European whites [2, 4]. Coronary artery disease (CAD) is the leading cause of premature CVD death in European whites and Asians; however, in Afro-Caribbean populations death from stroke is the leading cause [5]. Although conventional risk factors contribute to the differences in CVD, they do not adequately explain the excess risk noted in Afro-Caribbean and Indian Asian populations [2]. However, there may be certain ethnic specific CVD risk factors [3, 6].

Afro-Caribbean and Indian Asian populations are under represented in epidemiological and cardiovascular research trials [7]. As a result, minority ethnic groups may be at risk of significant disadvantage across a range of health indicators. Reporting racial information is important since findings from European white populations may not necessarily be extrapolated to other ethnic groups, given the observed differences in CVD mortality.

Prior research has demonstrated a survival difference between white, black, and Hispanic ethnic groups undergoing exercise treadmill testing, even after adjustment for patient demographics, baseline electrocardiography abnormalities, CVD co-morbidities and risk factors, and exercise test findings [8]. Dobutamine stress echocardiography (DSE) is a widely accepted and useful non-invasive test for the diagnosis, risk stratification and prognosis of CAD [9–12], as well as having greater specificity and sensitivity for CAD diagnosis compared to exercise treadmill testing. In addition, DSE has provided important prognostic information among different age groups [13], in men and women [14], as well as in patients with diabetes [15] and renal disease [16]. However, despite the available evidence, no study has investigated the role of DSE in predicting outcome amongst Afro-Caribbean, Caucasian and Indian Asian patients or determined whether myocardial ischaemia and ischaemic burden have the same prognostic power in these different ethnic populations. Therefore the aim of this prospective cohort study was to evaluate the role of DSE in the prediction of non-fatal cardiac events (NFCE) and all cause mortality by ethnic group. We studied a large cohort of consecutive patients referred for DSE and evaluated outcomes in UK Afro-Caribbean, European whites and Indian Asian patients.

## Methods

### Study design and patients

The study population consisted of 6231 consecutive patients undergoing DSE for the evaluation of angina pectoris or shortness of breath on exertion between June 2006 to September 2011 in the outpatient setting. All patients were required to provide informed consent before testing and the local research ethics committee approved the study. Ethnicity was obtained through self-report using the 2001 UK Census categorisation for classifying ethnicity. Exclusion criteria included mixed ethnic groups, Chinese ethnicity, patients referred for viability assessment only, asymptomatic patients awaiting non-cardiac surgery, patients with severe valve disease, and patients who did not provide signed informed consent.

Before DSE, a structured history and medical record review was performed to document symptoms, medical history, medication use, cardiac risk factors, and previous cardiac events and procedures. The estimation of the pre-test probability of CAD was determined using previously described criteria, which included the presence and characteristics of chest pain, age, gender, and presence of greater than or equal to three CAD risk factors [17]. Patients were categorised into low, intermediate or high pre-test probability. For patients who underwent multiple DSE tests during this period, only the first DSE was considered in the analysis.

Follow-up data was obtained by investigators blinded to the DSE result and information was collated by contacting patients or a family member, general practitioners, and reviewing hospital records to inquire about interim hospital admissions, outpatient diagnosis of cardiovascular events, and deaths. The date of the last review or consultation was used to calculate the duration of follow-up through to June 2013.

### Dobutamine stress echocardiography

All patients recruited underwent DSE. The image quality obtained was interpretable in all patients (1296 [24.3 %] requiring contrast) and the entire cohort was used in data analysis. DSE was performed according to a standard protocol [18] with images acquired in the standard parasternal long- and short-axis and apical 2-, 3-, and 4-chamber views. The left ventricle (LV) was divided into a 17-segment model for qualitative analysis [19] and wall motion was scored on a 4-point scale (1, normal wall motion; 2, hypokinesis; 3, akinetic; and 4, dyskinetic) as is standard [18]. In patients with resting akinetic segments a biphasic response was used to indicate ischaemia. LV ejection fraction was calculated using biplane Simpson’s technique. LV mass was derived from two-dimensional motion-mode and indexed to height [20]. Results were classified as a normal response with an overall increase in wall motion or abnormal response. An abnormal response was described as the occurrence under stress of hypokinesia, akinesia or dyskinesia in one or more resting normal segments and/or worsening of wall motion in one or more resting hypokinetic segments [21]. In this way patients were categorised as non-ischaemic or ischaemic. The extent and location of inducible ischaemia were evaluated and a wall motion score index (WMSI) was calculated, both at rest and during stress. Patients were further categorised with low (1–3 ischaemic LV segments) or high (>3 ischaemic LV segments) ischaemic burden. Non-viable myocardium was defined as severely dysfunctional myocardium without change during DSE [22] and referred to as fixed wall motion abnormalities (WMA). The territory of myocardial ischaemia was described according to an overlap model as previously described [23]: Left anterior descending artery (LAD) – anterior wall, anteroseptum, mid and apical inferoseptum; Circumflex artery (Cx) – mid inferolateral wall, basal and mid lateral wall; Right coronary artery (RCA)/Cx artery – inferior wall, basal inferolateral wall, basal inferoseptum.

### End point definition

The principal end-point of interest for this analysis was non-fatal cardiac events (NFCE) and secondarily death from any cause, with patients censored at the time of the last follow-up. A NFCE was defined as hospitalisation for myocardial infarction, unstable angina, and time to coronary revascularisation procedures, defined either as coronary artery bypass graft surgery or percutaneous coronary intervention. Hospitalisations were identified by means of the principle discharge diagnosis. For patients with multiple events, only the first event was considered.

### Data analysis

Continuous variables are expressed as mean ± SD and categorical variables as n (%). We used chi-square tests for discrete variables and one-way analysis of variance tests for continuous variables to test for differences in demographics, risk factors and DSE test results between ethnic groups and between participants with and without end point events. To describe the frequency of NFCE and all-cause mortality according to time since DSE, Kaplan-Meier cumulative event curves were constructed and compared using the log-rank test with a *P* value <0.05 considered statistically significant. The data were stratified according to A) ischaemic and non-ischaemic patients and B) non-ischaemic (0 segments), low ischaemic burden (1–3 ischaemic LV segments) and high ischaemic burden (>3 ischaemic LV segments) patients for NFCE and all-cause mortality. Event rates were calculated and expressed as percent per year. We used Cox proportional-hazards regression to estimate hazard ratios and corresponding 95 % confidence intervals (CI) for NFCE and all-cause mortality for each individual ethnic group and computed the C statistic (area under the receiver operator curve) as a measure of the incremental value of DSE beyond that provided by standard risk factors.

All models were adjusted for age, gender, smoking history, diabetes, hypercholesterolemia, hypertension, prior myocardial infarction, prior revascularization and use of lipid lowering or anti-hypertensive medication. All analyses were conducted using the statistical package for social sciences (SPSS 21 release version of SPSS for Windows; SPSS Inc., Chicago IL, USA).

## Results

### Study cohort

**Fig 1:**
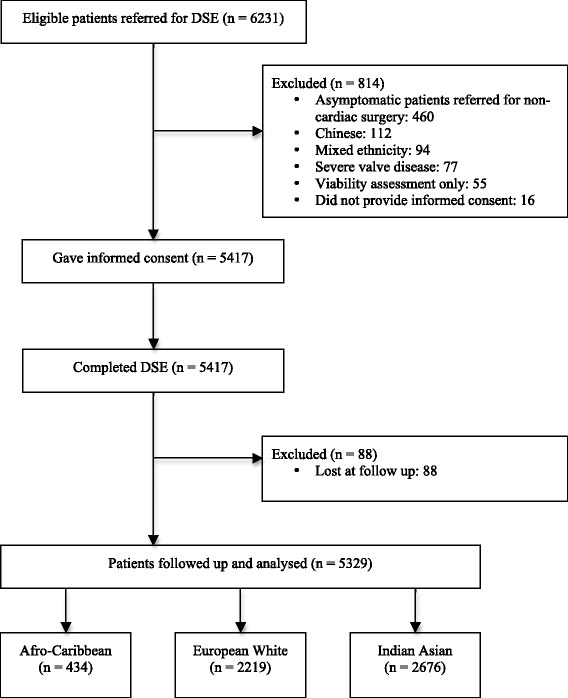
Study flow diagram

Of the 6231 patients referred for DSE, 814 did not meet inclusion criteria and 88 patients were lost at follow-up and therefore excluded from the final analysis. The remaining 5329 patients of which 434 (8.1 %) were Afro-Caribbean, 2219 (41.6 %) were European white and 2676 (50.2 %) were Indian Asian comprised the final study cohort (Fig. 1). Afro-Caribbean and Indian Asian patients had been resident in the UK for 38 ± 15 and 41 ± 17, respectively, and European whites were born in the UK. The majority of DSE requests were due to suspected angina pectoris (79.7 %) and the remainder were due to shortness of breath on exertion (20.3 %). The baseline characteristics of the study cohort varied significantly among the three ethnic groups, as shown in Table 1. Briefly, European white patients were significantly older than both Afro-Caribbean and Indian Asian patients. The presence of hypertension, diabetes mellitus, hypercholesterolemia and previous percutaneous coronary intervention was significantly greater in Indian Asians compared to Afro-Caribbeans and European whites. Family history of cardiovascular disease and smoking history was significantly greater in European whites compared to Afro-Caribbeans and Indian Asians. The low, intermediate and high pre-test probability of CAD did not significantly (*p* = 0.415) differ between ethnic groups (Table 1).

**Table 1:** Baseline demographic characteristics, risk factors and echocardiography measures according to ethnic group

Characteristics		Afro-Caribbean (*n* = 434)	European White (*n* = 2219)	Indian Asian (*n* = 2676)	*P* Value
Demographics					
	Age (yrs)	63.1 ± 12.6	66.8 ± 12.9	64 ± 11.5	<0.001
	Male gender	196 (45.2)	1144 (51.6)	1395 (52.1)	0.079
	Height (cm)	169.8 ± 8.7	170.4 ± 8.9	166.6 ± 9.8	<0.001
	Weight (kg)	83.4 ± 16.5	81.4 ± 15.8	79 ± 14.5	<0.001
	Body mass index (kg · m^2^)	29 ± 6	28 ± 5.2	28.5 ± 4.9	<0.001
	Body surface area (m^2^)	1.94 ± 0.2	1.93 ± 0.2	1.87 ± 0.2	<0.001
History					
	Hypertension	247 (56.9)	1228 (55.3)	1624 (60.7)	0.001
	Diabetes mellitus	102 (23.5)	451 (20.3)	880 (32.9)	<0.001
	Hypercholesterolemia	203 (46.8)	967 (43.6)	1441 (53.8)	<0.001
	Family history of CVD	91 (21)	586 (26.4)	644 (24.1)	0.026
	Prior myocardial infarction	30 (6.9)	204 (9.2)	256 (9.6)	0.204
	Prior PCI	75 (17.3)	450 (20.3)	732 (27.4)	0.009
	Prior CABGS	46 (10.6)	237 (10.7)	359 (13.4)	0.958
	Smoking history				<0.001
	Never smoked	350 (80.6)	1411 (63.6)	2337 (87.3)	
	Ex-smoker	61 (14.1)	553 (24)	220 (8.2)	
	Current smoker	23 (5.3)	255 (11.1)	119 (4.4)	
Pre-test probability of coronary artery disease					0.415
	Low	137 (31.6)	616 (27.8)	723 (27)	
	Intermediate	164 (37.8)	872 (39.3)	1064 (39.8)	
	High	133 (30.6)	731 (32.9)	889 (33.2)	
NYHA functional class symptom status					<0.001
	NYHA functional class II	411 (94.7)	1962 (88.4)	2317 (86.6)	
	NYHA functional class III	23 (5.3)	257 (11.6)	359 (13.4)	
Canadian Cardiovascular Society angina classification					0.001
	Class I	255 (58.8)	1096 (49.4)	1338 (50)	
	Class II	153 (35.3)	874 (39.4)	1046 (39.1)	
	Class III	26 (6)	249 (11.2)	292 (10.9)	
Long term cardiac medication					
	ACE inhibitor	155 (35.7)	817 (36.8)	959 (35.8)	0.759
	Angiotensin II receptor antagonist	79 (18.2)	410 (18.5)	545 (20.4)	0.202
	Aspirin	244 (56.2)	1211 (54.6)	1529 (57.1)	0.194
	Beta blockers	187 (43.1)	962 (43.4)	1136 (42.5)	0.815
	Calcium antagonists	140 (32.3)	662 (29.8)	847 (31.7)	0.319
	Diuretic	104 (24)	491 (22.1)	605 (22.6)	0.707
	Lipid-lowering agents	273 (62.9)	1478 (66.6)	1812 (67.7)	0.139
	Nitrates	61 (14.1)	323 (14.6)	403 (15.1)	0.806
	Warfarin	20 (4.6)	157 (7.1)	159 (5.9)	0.085
	At least 1 anti-anginal medication	285 (65.7)	1404 (63.3)	1721 (64.3)	0.563
Baseline Echocardiography Data					
	LVESD (cm)	2.9 ± 0.7	3.2 ± 0.6	3.1 ± 0.6	0.009
	LVEDD (cm)	4.48 ± 0.6	4.5 ± 0.5	4.38 ± 0.4	<0.001
	LV ejection fraction (%)	56.6 ± 8.9	56.2 ± 8.9	56.9 ± 7.9	0.023
	Maximal LVEDD Wall Thickness (cm)	1.19 ± 0.33	1.11 ± 0.21	1.12 ± 0.24	<0.001
	Left atrial size (mm)	38 ± 15	37 ± 11	37 ± 17	0.781
	Left ventricular mass (g)	182.8 ± 35.2	169.7 ± 38.6	153.3 ± 34.8	<0.001
	Left ventricular mass index (g · m^−1^)	103.8 ± 21.2	94.1 ± 23.5	89.9 ± 19.4	<0.001
	Mitral E/A	1.21 ± 0.4	1.22 ± 0.3	1.22 ± 0.4	0.72
	Mitral E Deceleration (ms)	202 ± 55	209 ± 69	203 ± 63	0.623
	Mitral E/Ea	9.6 ± 3.9	9.5 ± 4.3	9.6 ± 4.1	0.875
	Mitral Annular Calcification	14 (3.2)	73 (3.3)	130 (4.9)	0.014
	Mitral Regurgitation	54 (12.4)	315 (14.2)	384 (14.3)	0.564
	Aortic Stenosis	15 (3.5)	71 (3.2)	48 (1.8)	0.003
	Aortic Regurgitation	14 (3.2)	66 (3)	62 (2.3)	0.274
Dobutamine stress echocardiography test					
	Baseline heart rate (b · min^−1^)	69.6 ± 16.1	69.3 ± 18.8	71.1 ± 15.1	0.001
	Peak heart rate (b · min^−1^)	137.1 ± 21.7	131.8 ± 22.7	136.3 ± 19.1	<0.001
	Target heart rate achieved	358 (82.5)	1842 (83)	2231 (83.4)	0.943
	Baseline sBP (mmHg)	133.3 ± 24.1	131.2 ± 24.8	132.9 ± 24.5	0.039
	Peak sBP (mmHg)	160.3 ± 83	147.8 ± 31.5	151.1 ± 31.9	<0.001
	Baseline dBP (mmHg)	71.7 ± 18.3	71.3 ± 22.9	70.7 ± 19.6	0.498
	Peak dBP (mmHg)	75.1 ± 18.7	72.5 ± 17.6	74.5 ± 18.1	<0.001
	Resting wall motion score index	1.03 ± 0.1	1.05 ± 0.13	1.04 ± 0.11	0.006
	Peak wall motion score index	1.06 ± 0.13	1.09 ± 0.16	1.08 ± 0.15	0.001
	Fixed wall motion abnormality	55 (12.7)	377 (17)	427 (16)	0.078
	New wall motion abnormality	69 (15.9)	485 (21.9)	620 (23.2)	0.003
Number of ischaemic LV segments					<0.001
	0 LV segments	365 (84.1)	1734 (78.1)	2056 (76.8)	
	1-3 LV segments	61 (14.1)	398 (17.9)	555 (20.7)	
	>3 LV segments	8 (1.8)	87 (3.9)	65 (2.4)	
Outcome					
	Non-fatal cardiac event	55 (12.7)	363 (16.4)	431 (16.1)	0.149
	All-cause mortality	108 (24.9)	564 (25.4)	693 (25.9)	0.870

Note: *CVD* Cardiovascular disease, *PCI* Percutaneous coronary intervention, *CABGS* Coronary artery bypass graft surgery, *NYHA* New York Heart Association, *ACE* Angiotensin converting enzyme, *LVESD* Left ventricular end systolic dimension, *LVEDD* Left ventricular end diastolic dimension, *LV* Left ventricle, *sBP* systolic blood pressure, *dBP* diastolic blood pressure

Baseline atrial fibrillation was present in 82 (1.5 %) patients, and 116 (2.2 %) had left bundle branch block. Atrial fibrillation induced by DSE occurred in 27 (0.5 %) patients, and non-sustained ventricular tachycardia in 2 (0.04 %) patients. None of the patients required intravenous beta-blocker to reverse the effects of dobutamine or treat arrhythmias. Long-term cardiac medication was similar among all groups and the proportion of patients prescribed anti-anginal (defined as any treatment alone or in combination of beta-blockers, calcium antagonists, or nitrates) medication was similar between groups (Table 1). The Canadian Cardiovascular Society angina classification was similar between European white and Indian Asians, but significantly different from Afro-Caribbeans.

Importantly, there were significant differences in baseline cardiac function, structure and geometry between ethnic groups. Afro-Caribbean patients had a significantly lower LV end systolic diameter and significantly greater LV end diastolic diameter (LVEDD) maximal wall thickness, LV mass, and LV mass index compared to European white and Indian Asian patients (Table 1). Indian Asian patients had a significantly smaller LVEDD compared to Afro-Caribbean and European white patients and a significantly greater LV ejection fraction compared to European white patients (Table 1). The cardiovascular risk profile was less favourable in patients whom went onto have a NFCE and among those who died during follow-up, as shown in Table 2.

**Table 2:** Baseline demographic characteristics and risk factors according to non-fatal cardiac events and all-cause mortality in all patients

Parameter		No non-fatal cardiac event (*n* = 4480)	Non-fatal cardiac event (*n* = 849)	*P* Value	Survived (*n* = 3964)	All-cause mortality (*n* = 1365)	*P* Value
Demographics							
	Age (yrs)	64.4 ± 12.4	68.9 ± 11	<0.001	65.1 ± 11.8	65.1 ± 13.5	0.948
	Male gender	2315 (51.7)	420 (49.5)	0.451	2124 (53.6)	611 (44.8)	<0.001
	Ethnic group			0.149			0.870
	Black	379 (8.5)	55 (6.5)		326 (8.2)	108 (7.9)	
	European White	1856 (41.4)	363 (42.8)		1655 (41.8)	564 (41.3)	
	Indian Asian	2245 (50.1)	431 (50.8)		1983 (50)	693 (50.8)	
	Weight (kg)	79.3 ± 15.3	80.6 ± 15.2	0.027	78.9 ± 15.2	80.9 ± 15.3	<0.001
	Body mass index (kg · m^2^)	28.3 ± 5.1	28.3 ± 5.1	0.659	28 ± 5	28.4 ± 5.3	0.014
	Body surface area (m^2^)	1.88 ± 0.2	1.9 ± 0.2	0.003	1.88 ± 0.2	1.91 ± 0.2	<0.001
	Systolic blood pressure (mmHg)	132 ± 33	132 ± 39	0.934	131 ± 25	132 ± 25	0.356
	Diastolic blood pressure (mmHg)	70 ± 18	76 ± 30	<0.001	70 ± 20	71 ± 21	0.381
	LV ejection fraction (%)	56.9 ± 8.3	55.1 ± 9.2	<0.001	57.2 ± 7.8	54.9 ± 9.9	<0.001
History							
	Hypertension	2547 (56.9)	552 (65)	<0.001	2310 (58.3)	789 (57.8)	0.913
	Diabetes mellitus	1154 (25.8)	279 (32.9)	<0.001	1043 (26.3)	390 (28.6)	0.104
	Hypercholesterolemia	2098 (46.8)	513 (60.4)	<0.001	1886 (47.6)	725 (53.1)	<0.001
	Family history of CVD	1072 (23.9)	249 (29.3)	0.001	926 (23.4)	395 (28.9)	<0.001
	Prior myocardial infarction	394 (8.8)	96 (11.3)	0.020	356 (9)	134 (9.8)	0.346
	Prior PCI	1034 (23.1)	223 (26.3)	0.124	940 (23.7)	317 (23.2)	0.784
	Prior CABG	508 (11.3)	134 (15.8)	<0.001	431 (10.9)	211 (15.5)	<0.001
	Smoking history			0.024			0.503
	Non-smoker	3474 (77.5)	629 (74.1)		3047 (76.9)	1056 (77.4)	
	Ex-smoker	690 (15.4)	139 (16.4)		628 (15.8)	201 (14.7)	
	Current smoker	316 (7.1)	81 (9.5)		289 (7.3)	108 (7.9)	
NYHA functional class symptom status				<0.001			<0.001
	NYHA functional class II	4063 (90.7)	627 (73.9)		3665 (92.5)	1025 (75.1)	
	NYHA functional class III	417 (9.3)	222 (26.1)		299 (7.5)	340 (24.9)	
CCS angina classification				<0.001			<0.001
	Class I	2689 (60)	0 (0)		2202 (55.5)	487 (35.7)	
	Class II	1402 (31.3)	671 (79)		1524 (38.4)	549 (40.2)	
	Class III	389 (8.7)	178 (21)		238 (6)	329 (24.1)	
Long term cardiac medication							
	ACE inhibitor	1632 (36.4)	299 (35.2)	0.551	1443 (36.4)	488 (35.8)	0.648
	Angiotensin II receptor antagonist	861 (19.2)	173 (20.4)	0.404	753 (19)	281 (20.6)	0.206
	Aspirin	2578 (57.5)	406 (47.8)	<0.001	2176 (54.9)	808 (59.2)	0.006
	Beta blockers	1965 (43.9)	320 (37.7)	0.001	1674 (42.2)	611 (44.8)	0.109
	Calcium antagonists	1389 (31)	260 (30.6)	0.880	1222 (30.8)	427 (31.3)	0.771
	Diuretic	1005 (22.4)	195 (23)	0.714	857 (21.6)	343 (25.1)	0.007
	Lipid-lowering agents	3027 (67.6)	536 (63.1)	0.017	2614 (65.9)	949 (69.5)	0.017
	Nitrates	689 (15.4)	98 (11.5)	0.004	581 (14.7)	206 (15.1)	0.724
	Warfarin	271 (6)	65 (7.7)	0.073	245 (6.2)	91 (6.7)	0.530

Note: *CVD* Cardiovascular disease, *PCI* Percutaneous coronary intervention, *CABGS* Coronary artery bypass graft surgery, *NYHA* New York Heart Association, *ACE* Angiotensin converting enzyme

### Clinical outcomes

The mean follow-up time was 4.6 ± 1.3 years (Afro-Caribbean: 4.6 ± 1.2 years; European white 4.4 ± 1.4 years; Indian Asians 4.8 ± 1.2 years). NFCE occurred in 849 (15.9 %) patients overall and were noted in 12.7 % (55 events) Afro-Caribbean patients, 16.4 % (363 events) in European white patients and 16.1 % (431 events) in Indian Asians. All-cause mortality occurred in 1365 (25.6 %) of patients (24.9 % in Afro-Caribbean patients, 25.4 % in European white patients and 25.9 % in Indian Asians). There were no significant differences between ethnic groups regarding NFCE and all-cause mortality.

DSE was completed in all patients and the level of agreement; kappa between the two sonographers was 0.84. Consensus was obtained in discordant cases and 90,593 left ventricular segments were analysed. In total 3645 (68.4 %) patients had a normal study, 1174 (22 %) patients developed a new or worsening WMA (ischaemic response) during their DSE, and 859 (16.1 %) had fixed WMA’s. Of the patients with fixed WMA’s, 349 (40.6 %) developed a new or worsening WMA during DSE. The territory of myocardial ischaemia did not significantly differ between groups (LAD, *p* = 0.821; Cx, *p* = 0.748; RCA, *p* = 0.975).

During the follow-up period, 958 (18%) patients (85 [19.6 %] Afro-Caribbean, 366 [16.5 %] European white, 507 [18.9 %] Indian Asian) underwent coronary angiography within 29 ± 2.2 days of DSE. Of these patients, 561 (58.6 %) had inducible ischaemia during DSE. In total, 530 (9.9 %) patients had significant (defined as ≥70 % coronary lumen stenosis by visual determination in ≥1 coronary artery) CAD (47 [10.8 %] Afro-Caribbean, 213 [9.6 %] European white, 270 [10.1 %] Indian Asian patients). The resulting sensitivity, specificity, positive and negative predictive values for DSE in detecting significant CAD were 93.6, 84.8, 88.4 and 91.4 %, respectively. There were no significant differences between the ethnic groups in the proportion of patients who underwent coronary angiography (*p* = 0.181) or the proportion of patients who had significant coronary disease (*p* = 0.781).

The NFCE rate for all patients without ischaemia was 2.3 % per year, increasing to 4.8 % for patients with fixed WMA’s, 7.3 % per year for those with 1–3 ischaemic segments and highest among those with >3 ischaemic segments (10.1 % per year). Patients with any ischaemia during DSE had a cardiac event rate of 7.7 % per year, suggesting that a positive DSE was associated with 90 extra NFCE per 100 person years of follow-up.

The all-cause mortality event rate for all patients without ischaemia was 4 % per year, increasing to 7.9 % for those with fixed WMA’s, 10.5 % per year for those with 1–3 ischaemic segments and highest among those with >3 ischaemic segments (16 % per year). Patients with any ischaemia during DSE had a mortality event rate of 11.2 % per year, suggesting that a positive DSE was associated with 132 extra deaths per 100 person years of follow-up.

### Dobutamine stress echocardiography as a predictor of outcome

**Fig 2:**
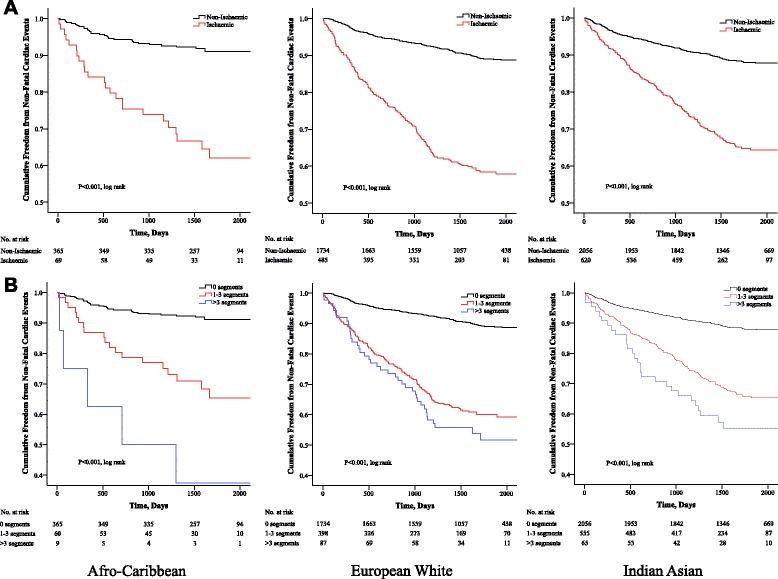
Kaplan-Meier hazard curves for the cumulative survival and freedom from non-fatal cardiac events in each ethnic group. Kaplan-Meier hazard curves dichotomized according to myocardial ischaemia (**a**) and number of ischaemic LV segments (**b**)

**Fig 3:**
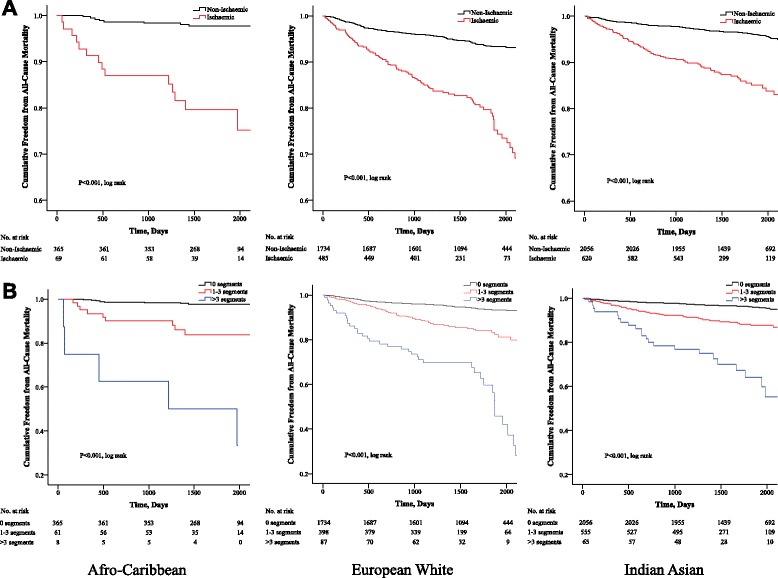
Kaplan-Meier hazard curves for the cumulative survival and freedom from all-cause mortality in each ethnic group. Kaplan-Meier hazard curves dichotomized according to myocardial ischaemia (**a**) and number of ischaemic LV segments (**b**)

Figure 2 and Fig. 3 show the unadjusted Kaplan-Meier cumulative event curves for NFCE and all-cause mortality, respectively, dichotomized according to myocardial ischaemia (a) and number of ischaemic LV segments (b). The differences amongst these curves were significant (*P* < 0.001) and illustrates that myocardial ischaemia and greater ischaemic burden translate into significantly worse outcome.

Table 3 and Additional file 1: Table S1 show the risk of NFCE and all-cause mortality respectively in each of the three ethnic groups, adjusted for standard risk factors including age, gender, hypertension, diabetes mellitus, hypercholesterolemia, family history of CVD, smoking history, prior myocardial infarction, prior revascularization and use of lipid lowering or anti-hypertensive medication. Among the three ethnic groups, ischaemia on DSE was associated with 2 to 2.5 times the risk of NFCE and 1.2 to 1.4 times the risk for all-cause mortality. For each ethnic group, the risk associated with a NFCE and all-cause mortality increased as the burden of myocardial ischaemia increased. Importantly, peak WMSI was the strongest independent predictor in all-ethnic groups for both NFCE and all-cause mortality. All adjusted hazard ratios for DSE parameters are significant (*P* < 0.038), for NFCE and all-cause mortality.

**Table 3:** Risk of non-fatal cardiac events associated with dobutamine stress test result in three ethnic groups

		Afro-Caribbean		European White		Indian Asian	
Parameter		HR (95 % CI)	*P*	HR (95 % CI)	*P*	HR (95 % CI)	*P*
Age (yrs)		1.025 (1.000-1.051)	0.050	1.020 (1.009-1.031)	0.001	1.018 (1.008-1.027)	0.002
Male gender		1.366 (0.793-2.353)	0.262	0.967 (0.534-1.752)	0.913	0.870 (0.720-1.052)	0.152
Hypertension		2.201 (1.089-4.449)	0.028	0.910 (0.721-1.149)	0.428	1.040 (0.823-1.315)	0.714
Diabetes mellitus		1.038 (0.549-1.963)	0.909	1.118 (0.865-1.445)	0.393	2.652 (2.538-2.790)	<0.001
Hypercholesterolemia		0.831 (0.434-1.590)	0.575	1.773 (1.624-1.959)	0.019	1.347 (1.073-1.691)	0.010
Family history of CVD		1.177 (0.582-2.380)	0.650	1.350 (1.078-1.691)	0.009	0.906 (0.717-1.145)	0.407
Prior myocardial infarction		1.311 (0.552-3.644)	0.164	1.126 (0.792-1.601)	0.509	1.028 (0.743-1.422)	0.868
Prior PCI		0.520 (0.223-1.211)	0.130	0.826 (0.631-1.081)	0.164	0.981 (0.793-1.214)	0.863
Prior CABG		2.496 (1.199-5.198)	0.015	1.043 (0.764-1.424)	0.790	0.781 (0.590-1.034)	0.085
Smoking history			0.565		0.252		0.658
	Non-smoker	1 (reference)		1 (reference)		1 (reference)	
	Ex-smoker	0.738 (0.263-2.076)		0.871 (0.671-1.130)		1.028 (0.709-1.491)	
	Current smoker	1.260 (0.365-4.347)		1.375 (1.004-1.885)		1.326 (0.867-2.027)	
ACE inhibitor		0.963 (0.507-1.830)	0.909	0.989 (0.780-1.253)	0.925	1.029 (0.833-1.272)	0.788
Angiotensin II receptor antagonist		1.090 (0.513-2.313)	0.823	1.099 (0.822-1.469)	0.524	1.158 (0.909-1.474)	0.236
Beta blockers		0.965 (0.532-1.751)	0.906	0.805 (0.644-1.008)	0.059	0.766 (0.625-0.939)	0.010
Calcium antagonists		1.532 (0.838-2.801)	0.166	0.871 (0.685-1.108)	0.261	0.985 (0.801-1.212)	0.888
Lipid-lowering agents		0.969 (0.509-1.843)	0.922	0.715 (0.579-0.883)	0.002	0.868 (0.701-1.073)	0.191
Fixed wall motion abnormality		0.879 (0.116-1.240)	0.109	0.933 (0.628-1.384)	0.729	0.827 (0.555-1.234)	0.353
Resting wall motion score index		0.749 (0.220-9.947)	0.304	1.489 (1.200-1.848)	0.008	1.991 (1.985-1.997)	0.003
Peak wall motion score index		2.273 (2.114-17.483)	0.038	3.092 (3.026-6.306)	<0.001	3.643 (3.254-12.033)	<0.001
New wall motion abnormality		2.026 (1.121-2.351)	<0.001	2.105 (1.166-2.251)	<0.001	2.490 (1.340-4.620)	<0.001
Number of Ischaemic LV Segments			<0.001		<0.001		<0.001
	0 LV segments	1 (reference)		1 (reference)		1 (reference)	
	1-3 LV segments	1.092 (1.036-1.238)		1.171 (1.121-1.242)		1.62 (1.44-4.18)	
	>3 LV segments	2.192 (2.146-4.238)		2.803 (2.567-5.138)		3.040 (2.68-7.70)	

Note: *CVD* Cardiovascular disease, *PCI* Percutaneous coronary intervention, *CABGS* Coronary artery bypass graft surgery, *ACE* Angiotensin converting enzyme

Table 4 shows the C-statistic for the prediction of NFCE and all-cause mortality according to ethnic group, calculated on the basis of the standard risk factors alone and on the basis of the standard risk factors in addition to DSE parameters. The C-statistic for the prediction of NFCE and all-cause mortality was greater when DSE parameters were added to standard risk factors. These increases were statistically significant for each ethnic group indicating an improvement in discrimination.

**Table 4:** C-statistic for risk factors alone and for risk factors plus dobutamine stress test results to predict non-fatal cardiac events and all-cause mortality

Ethnic Group	Non-fatal cardiac event			All-cause mortality		
	C-statistic for risk factors alone	C-statistic for risk factors plus DSE test results	P value	C-statistic for risk factors alone	C-statistic for risk factors plus DSE test results	P value
Afro-Caribbean	0.67	0.74	0.009	0.6	0.72	0.004
European White	0.68	0.76	<0.001	0.61	0.72	<0.001
Indian Asian	0.64	0.74	<0.001	0.57	0.69	<0.001
Total	0.64	0.74	<0.001	0.61	0.68	<0.001

## Discussion

We examined the predictive value of DSE in a multiethnic UK population. We found that DSE is a strong and independent predictor of NFCE and all-cause mortality irrespective of ethnicity. The risk of NFCE and all-cause mortality was associated with the burden of myocardial ischaemia, as assessed by the peak WMSI and the number of ischaemic segments during DSE. The addition of DSE to standard risk factor models including age, gender, hypertension, diabetes mellitus, hypercholesterolemia, family history of CVD, smoking history, prior myocardial infarction, prior revascularization and use of lipid lowering or anti-hypertensive medication for the prediction of NFCE and all-cause mortality significantly increased the C statistic, an order of magnitude comparable to that observed with coronary calcium scoring in different ethnic populations [24, 25]. Importantly, DSE parameters contributed to the risk of both NFCE and all-cause mortality in three major ethnic groups independently of other risk factors.

The prognostic value of DSE has been previously reported in large studies in patients with various pre-test probabilities [9, 13, 16, 26–31]. Ischaemic burden and severe wall motion abnormalities during stress were independently associated with events in the present study, findings which previous research have recognised that indicate worse prognosis [9, 13]. However, previous analysis has not attempted to evaluate the predictive value of DSE in different ethnic groups. Importantly, in all three ethnic groups studied, the addition of DSE parameters to clinical data improved the predictive power. This finding contrasts prior research, which demonstrated ethnic differences in the survival of patients undergoing exercise treadmill testing [8].

This study also demonstrated ethnicity related differences in the function, structure and geometry of the left ventricle at rest. Afro-Caribbean patients demonstrated greater concentric cardiac remodelling with significantly greater maximal LV wall thickness and LV mass compared to European white and Indian Asian patients. A greater LV wall thickness has been shown previously in Afro-Caribbean populations compared to other ethnic groups and is associated with an increased mortality risk [32]. Furthermore, an increased LV mass has been shown to be a powerful independent predictor for CVD morbidity and mortality in individuals previously free of clinical cardiovascular disease [33]. Treatment to control modifiable risk factors in Afro-Caribbean populations may reduce cardiac remodelling [34]. Indian Asian patients had a significantly smaller LV cavity and the smallest LV mass compared to Afro-Caribbean and European white patients. Other studies support these findings [34–36]. Indexing LV mass did not attenuate the observed difference. A decreased LV cavity and smaller LV mass may lead to an increase in LV wall stress and myocardial oxygen demand, which may increase the vulnerability to myocardial ischaemia [34]. This was a specific cohort of patients referred for DSE. These differences may contribute to the elevated CVD risk seen in Afro-Caribbean and Indian Asian ethnic groups. It is important to note that European white patients were significantly older than both Afro-Caribbean and Indian Asian patients. Therefore, for an age matched cohort the prevalence of CVD may be significantly different between ethnic groups.

Angiographic comparisons of CAD between Caucasians and Asians have reported similar findings to the present study. Dhawan and Bray [37] reported no difference in the severity or extent of CAD between Caucasians and Asians. As shown in our study, there was a significant difference in age between groups, with Asians being significantly younger than Caucasians. In a recent study including 279,256 patients, South Asians were younger, had more extensive coronary artery disease and greater prevalence of major risk factors, particularly diabetes compared to Caucasians [38]. However, as shown in our study, when correcting for these differences, outcome was similar between South Asians and Caucasians. Similar to our study, angiographic differences in CAD were minimal among African American compared to white patients, with a similar distribution of coronary vessel disease and mean stenosis score [39].

Disproportionate rates of heart disease are seen in racial and ethnic minority populations. The ability to reliably risk stratify populations at greater risk of adverse cardiac events is therefore of great importance. Recently, cardiovascular screening demonstrated the potential to reduce ethnic health inequalities [40]. In our study Indian Asians had a significantly greater prevalence of hypertension, diabetes mellitus, and hypercholesterolemia compared to Afro-Caribbeans and European whites. However, a significantly greater proportion of Indian Asians had previous coronary intervention compared to Afro-Caribbeans and European whites. In addition, Indian Asian and European whites had greater Canadian Cardiovascular Society angina classification compared to Afro-Caribbeans. Irrespective of differences in risk factors, the results of this study indicate that DSE adds incremental value in estimating the probability of cardiac events and all-cause mortality in three major ethnic groups and is therefore a valuable tool in the assessment of CAD.

Our study has limitations. This was a prospective observational study from a single centre. Patients recruited into our study were referred for a clinically indicated DSE and there is the potential for referral bias and high pre-test probability related to a higher prevalence of co-morbidities and symptoms. Only 18.9 % on the study populations underwent coronary angiography within 1-month of DSE, which may bias test sensitivity and specificity. Medication listed refers to treatment at time of DSE and changes in medication over the follow-up period were not taken into account. Due to difficulties in accurately determining the cause of death by reviewing death certificates or medical records, all-cause mortality was selected as a more objective and unbiased end point [41]. Notwithstanding these limitations, the present study is consistent with earlier work and extends our knowledge in different ethnic populations.

## Conclusions

In a multiethnic UK cohort, DSE added incremental value to the prediction of NFCE and all-cause mortality over that of standard CVD risk factors in Afro-Caribbean, European white and Indian Asian patients.

## Additional file

Additional file 1: Table S1. Risk of all-cause mortality associated with dobutamine stress test result in three ethnic groups (DOCX 60 kb)
