# Supplementary material for: The prognostic value of dobutamine stress echocardiography amongst British Indian Asian and Afro-Caribbean patients: a comparison with European white patients
Source: Cardiovasc Ultrasound. 2015 Aug 6;13:36. doi: 10.1186/s12947-015-0028-1 (PMC4527129; doi:10.1186/s12947-015-0028-1)
Supplement: Additional file 1: Table S1. — Risk of all-cause mortality associated with dobutamine stress test result in three ethnic groups (DOCX 60 kb) [file 12947_2015_28_MOESM1_ESM.docx]

The Prognostic Value of Dobutamine Stress Echocardiography Amongst British Indian Asian and Afro-Caribbean Patients: A Comparison with European White Patients.

International Journal of Cardiovascular Imaging.

Jamie M O’Driscoll, Claire Rossato, Paula Gargallo-Fernandez, Marco Araco, Dimitrios Giannoglou, Sanjay Sharma, and Rajan Sharma.

**Address for correspondence:** Dr Rajan Sharma, Department of Cardiology, St George’s Healthcare NHS Trust, Blackshaw Road, Tooting, London, SW17 0QT. E-mail: rajan.sharma@stgeorges.nhs.uk; Telephone: +44 (0)2087250286; Fax: +44 (0)2087254402. There are no relationships with industry.

| Supplementary Table 1: Risk of all-cause mortality associated with dobutamine stress test result in three ethnic groups. | | | | | | | | |
| --- | --- | --- | --- | --- | --- | --- | --- | --- |
|  | |  | Afro-Caribbean | | European White | | Indian Asian | |
| Parameter | | | HR (95% CI) | *P* | HR (95% CI) | *P* | HR (95% CI) | *P* |
| Age (yrs) | | | 1.059 (1.017-1.102) | 0.025 | 1.010 (1.002-1.018) | 0.010 | 1.051 (1.034-1.069) | 0.002 |
| Male gender | | | 1.068 (0.730-1.560) | 0.736 | 1.906 (1.064-3.416) | 0.030 | 1.014 (0.874-1.177) | 0.850 |
| Hypertension | | | 1.100 (1.030-1.630) | 0.030 | 0.867 (0.717-1.048) | 0.140 | 1.034 (0.861-1.243) | 0.720 |
| Diabetes mellitus | | | 1.426 (0.879-2.314) | 0.151 | 1.070 (0.866-1.322) | 0.532 | 4.200 (4.160-7.900) | 0.001 |
| Hypercholesterolemia | | | 1.762 (0.650-2.621) | 0.446 | 1.437 (1.065-1.939) | 0.018 | 1.389 (1.155-1.670) | <0.001 |
| Family history of CVD | | | 0.743 (0.443-1.245) | 0.259 | 1.543 (1.018-2.340) | 0.041 | 0.949 (0.788-1.142) | 0.578 |
| Prior myocardial infarction | | | 1.250 (0.545-2.865) | 0.598 | 1.591 (1.424-1.824) | 0.002 | 0.968 (0.736-1.274) | 0.818 |
| Prior PCI | | | 0.584 (0.329-1.301) | 0.066 | 0.740 (0.589-0.931) | 0.036 | 0.576 (0.421-0.789) | 0.001 |
| Prior CABG | | | 0.682 (0.357-1.301) | 0.245 | 0.579 (0.403-0.834) | 0.003 | 1.025 (0.821-1.280) | 0.827 |
| Smoking history | | |  | 0.309 |  | 0.074 |  | 0.349 |
|  | Non-smoker | | 1 (reference) |  | 1 (reference) |  | 1 (reference) |  |
|  | Ex-smoker | | 0.590 (0.279-1.246) |  | 1.168 (0.889-1.533) |  | 0.949 (0.656-1.373) | 0.782 |
|  | Current smoker | | 1.644 (0.724-3.732) |  | 1.217 (0.982-1.509) |  | 1.019 (0.721-1.439) | 0.915 |
| ACE inhibitor | | | 1.320 (0.857-2.035) | 0.208 | 0.908 (0.755-1.092) | 0.305 | 0.872 (0.736-1.032) | 0.112 |
| Angiotensin II receptor antagonist | | | 0.604 (0.371-0.983) | 0.042 | 0.778 (0.615-0.984) | 0.036 | 1.047 (0.865-1.268) | 0.637 |
| Beta blockers | | | 1.013 (0.669-1.536) | 0.906 | 0.962 (0.808-1.145) | 0.662 | 1.013 (0.865-1.186) | 0.870 |
| Calcium antagonists | | | 1.483 (0.975-2.254) | 0.065 | 0.836 (0.690-1.012) | 0.066 | 0.986 (0.836-1.162) | 0.864 |
| Lipid-lowering agents | | | 0.837 (0.526-1.334) | 0.455 | 0.618 (0.174-0.731) | 0.003 | 0.713 (0.219-0.806) | 0.002 |
| Fixed wall motion abnormality | | | 0.687 (0.275-1.721) | 0.423 | 0.752 (0.540-1.049) | 0.093 | 1.023 (0.855-1.224) | 0.804 |
| Resting wall motion score index | | | 1.013 (0.019-5.339) | 0.157 | 1.079 (1.013-1.468) | 0.005 | 1.068 (1.048-1.094) | 0.021 |
| Peak wall motion score index | | | 4.982 (3.982-6.294) | <0.001 | 5.482 (2.385-12.597) | <0.001 | 5.654 (4.599-14.277) | <0.001 |
| New wall motion abnormality | | | 1.223 (1.095-1.559) | 0.001 | 1.230 (1.143-1.369) | <0.001 | 1.354 (1.77-1.708) | 0.003 |
| Number of Ischemic LV Segments | | |  | <0.001 |  | <0.001 |  | <0.001 |
|  | 0 LV segments | | 1 (reference) |  | 1 (reference) |  | 1 (reference) |  |
|  | 1-3 LV segments | | 1.027 (1.009-1.082) |  | 1.146 (1.111-1.191) |  | 1.62 (1.44-4.18) |  |
|  | >3 LV segments | | 1.675 (1.058-2.531) |  | 1.614 (1.464-1.812) |  | 3.040 (2.68-7.70) |  |
